# Fatty liver index relationship with biomarkers and lifestyle: result from RaNCD cohort study

**DOI:** 10.1186/s12876-023-02785-5

**Published:** 2023-05-22

**Authors:** Negin Kamari, Hawal Lateef Fateh, Mitra Darbandi, Farid Najafi, Mozhgan Moradi, Yahya Pasdar

**Affiliations:** 1 School of Nutritional Sciences and Food Technology, Kermanshah University of Medical Sciences, Kermanshah, Iran; 2 Nursing Department, Kalar Technical College, Sulaimani Polytechnic University, Sulaymaniyah, Iraq; 3 Research Center for Environmental Determinants of Health (RCEDH), Health Institute, Kermanshah University of Medical Sciences, Kermanshah, Iran; 4 Internal Medicine Department, School of Medicine, Kermanshah University of Medical Sciences, Kermanshah, Iran; 5 Department of Nutritional Sciences, School of Nutritional Sciences and Food Technology, Kermanshah University of Medical Sciences, Kermanshah, Iran

**Keywords:** Non-alcoholic fatty liver disease, Physical activity, Lifestyle, Fatty liver index

## Abstract

**Background:**

Lifestyle intervention can effectively treat patients with non-alcoholic fatty liver disease (NAFLD). The present study aimed to investigate the association between lifestyle factors with fatty liver index (FLI) in Iranian adults.

**Methods:**

This study enrolled 7114 subjects from the Ravansar Non-Communicable Diseases (RaNCD) cohort study in western Iran. To compute the FLI score, anthropometric measures, and a few non-invasive liver status indicators were used. Binary logistic regression models examined the association between FLI score and lifestyle.

**Results:**

Participants with FLI < 60 had a lower daily energy intake compared to those with FLI ≥ 60 (2740.29 vs. 2840.33 kcal/day, P = < 0.001). The risk of NAFLD in males with high socioeconomic status (SES) was 72% higher than in those with low SES (OR: 1.72; 95% CIs 1.42–2.08). An adjusted logistic regression model showed a significantly negative association between high physical activity and fatty liver index in both men and women. (OR: 0.44, p-value < 0.001 and OR: 0.54, p-value < 0.001, respectively). The odds of NAFLD in female participants with depression were 71% higher than in non-depressed participants (OR: 1.71, 95% CI: 1.06–2.64). Dyslipidemia and high visceral fat area (VFA) were also associated with a significant increase in the risk of NAFLD (P < 0.05).

**Conclusion:**

In our study, we found that good SES, high VFA, and dyslipidemia were associated with an increased risk of NAFLD. Conversely, high physical activity reduces the risk of NAFLD. Therefore, lifestyle modification may help improve liver function.

## Introduction

To be diagnosed with NAFLD, 5% hepatic steatosis must be present without large alcohol intake [1]. The world’s most prevalent chronic liver disease, NAFLD, has been linked to hepatocellular carcinoma and is a leading cause of the end-stage liver disease [2]. NAFLD prevalence varies from 17 to 35% worldwide [3]. Due to lifestyle changes, such as eating patterns and food, decreased physical activity, and an increase in obesity, NAFLD has become more prevalent in recent decades [2]. Metabolic disorders associated with fatty liver disease (MAFLD) is another definition that underlines the coexistence of hepatic steatosis and metabolic dysfunctions, which better reflects disease etiology and pathogenesis. Overweight or obese, the presence of T2DM, or evidence of metabolic dysregulation, characterized by the presence of at least two metabolic risk abnormalities (increased waist circumference, high blood pressure, hyperglyceridemia, reduced HDL-C concentration, glucose intolerance, or rise in HbA1c, HOMA-IR ≥ 2,5, increased hs-CRP level in serum) are its criteria [4]. As a treatment for NAFLD, clinicians emphasize the importance of an active lifestyle that includes regular exercise, a balanced diet, and weight reduction. Socioeconomic status (SES) is now recognized to affect NAFLD [5, 6]. Although the reason for SES being included as a risk factor for NAFLD has not been made clear, it may have a significant impact on people’s lives and living arrangements. Disparities in SES may impact on exercise habits, nutritional habits, and access to the healthcare system [7]. These potential causes suggest a relationship between SES and the main risk factors for NAFLD, such as insulin resistance, obesity, and lipid metabolic disease [5].

Patients with NAFLD are required to follow a treatment diet that demands a lifestyle change. The European Association for the Study of the Liver, the European Association for the Study of Diabetes, and the European Association for the Study of Obesity recommend three to five sessions per week of 150–200 min of moderately strenuous aerobic activity (EASL-EASD-EASO). They also suggest a Mediterranean diet, which decreases the consumption of foods high in sugar or saturated fat and increases those high in monounsaturated fatty acids and omega-3 fatty acids [8]. To successfully reduce steatosis, alleviate inflammation, or reverse liver fibrosis, participants must adhere to these strategies and lose 10% of their body weight [9]. However, over 50% of NAFLD patients have unsatisfactory adherence to therapy [10]. This may be partly explained in part by the patients’ typical lack of motivation to make changes [11]. Successful commitment to lifestyle changes resulted in an improved quantitative scale of depression than in the no adherent group [12–14]. Thus, the purpose of the present study was to investigate the association between lifestyle risk factors with fatty liver index in Iranian adults.

## Method

### Study design and participants

A cross-sectional was carried out as part of the Ravansar Non-Communicable Disease (RaNCD) cohort investigation. In summary, since 2014, RaNCD has been conducting population-based research to assess the prevalence of chronic illnesses and mortality in Ravansar, one of the Iranian provinces of Kermanshah. This study is a part of the PERSIAN mega-cohort study in Iran. In the western Iranian province of Kermanshah, near the Iraqi border, sits Ravansar, which has both urban and rural areas. It is home to around 50,000 people who are of Kurdish descent. Ten distinct geographic zones have been established in Iran for the PERSIAN cohort study. These locations have been carefully chosen based on local mortality morbidity, risk factors, and regional sickness patterns. Clinical research is primarily concerned with preventing non-communicable diseases, are responsible for around 70% of all illnesses and deaths in the country. The whole study team, which comprised a medical doctor, interviewers, laboratory technicians, executive managers, and receptionists, was chosen via a face-to-face interview procedure with the principal investigators. Demographics, food consumption, physical activity (PA), smoking status (never, presently, or previously smoked), Socio-Economic Status (weak, moderate, and good), dynamometer, anthropometric indices, and body composition were the main pieces of information that the study team gathered. The patient’s family history, present, and past medical conditions, and prescription drug use were all examined by the RaNCD doctor. A total of 2,933 people were excluded from the study, including those using liver medications (n = 529), those with CVDs (n = 1224), diabetes type 2 (n = 757), cancer (n = 50), Arthritis Romathoid (n = 158), and pregnant women (n = 96). Additionally, this research did not include missing data (n = 119). Seven thousand one hundred fourteen people eventually remained and were chosen for the study.

### Data collection

Demographic information, such as age, gender, marital status, economic standing, level of education, and history of smoking, sickness, and drug use, was gathered via face-to-face interviews with professional interviewers.

### Anthropometry

The participants’ heights were measured using an automated stadiometer BSM 370 (Biospace Co., Seoul, Korea) with a 0.1 cm precision while standing without shoes. The InBody 770 scale was used to assess weights when subjects were wearing the fewest number of clothing and without shoes (Inbody Co, Seoul, Korea). The weight and height were measured only once. The following formula was used to compute BMI: weight (kg)/height^2^ (meter).

### Healthy eating index 2015

Adult participants were selected using previously published semi-quantitative nationwide validated 118-item food frequency questionnaire (FFQ) surveys that included accurate and comprehensive dietary data [15]. The Krebs-Smith et al. technique and RaNCD FFQ data were used to compute the healthy eating index 2015 (HEI 2015) [16]. Whole fruits, full protein meals, whole vegetables, seafood and plant proteins, greens and beans, whole grains, dairy products, fatty acids, refined grains, salt, added sugars, and saturated fats are the thirteen components of the HEI 2015. These foods were divided into two groups using the food categories of sufficiency and moderation. If the usage is appropriate, the score is higher. An optimal diet should include whole fruits, whole protein meals, total vegetables, seafood, and plant proteins, greens and beans, whole grains, dairy products, and fatty acids. Conversely, a lower HEI 2015 score indicates that the last four components of a balanced diet—refined grains, salt, added sugars, and saturated fats—have been moderately consumed. The number of meals eaten is graded from 0 to 5 for the first six meals, then from 0 to 10 for the remaining meals. The sum of the scores for each item results in the total HEI score, ranges from 0 to 100. The first and fourth quartiles had the lowest and highest HEI scores, respectively.

### Biochemical factors

As per standard procedure for the RaNCD cohort study, blood samples were taken from the ante-brachial vein after 8–12 h of fasting. The blood concentrations of liver enzymes including Aspartate Transaminase (AST), Alanine Aminotransferase (ALT), and GGT as well as lipid profiles like LDL-C, TG, HDL-C, and total cholesterol were measured using enzymatic kits (Pars Azmun, Iran). Before analysis, the serum samples were centrifuged and kept in aliquots in cryotubes at -80 °C.

#### Fatty liver index

FLI was first presented by Bedogni et al. in 2006 with thirteen variables (including age, gender, ethanol intake, AST, ALT, GGT, WC, BMI, the sum of four skinfolds, Insulin, glucose, TG, and cholesterol), four of which remained as predictors in the equation.

FLI = (e^0.953*log e(triglycerides) + 0.139*BMI + 0.718*log e(ggt) + 0.053*waist circumference - 15.745^) / (1 + e ^0.953*log e(triglycerides) + 0.139*BMI + 0.718*log e(ggt) + 0.053*waist circumference - 15.745^) * 100.

see [17]

According to the area under the receiver operator characteristic curve (AUROC), the FLI’s fatty liver detection accuracy was 0.83 (95% CI: 0.825 to 0.842). [18]. The FLI scores are between 0 and 100. Thus, with a high degree of diagnostic accuracy, FLI scores of 30 and 60 indicated the absence or presence of fatty liver, respectively [17].

### Assessment of depression

Psychologists with expertise examined the research participants for symptomatic or non-symptomatic depression. A clinical examination and a self-administered questionnaire made up the screening process. Additionally, participants were asked whether they had previously received antidepressants.

### Assessment of physical activity

Physical activity levels were determined using (IPAQ) (which inquired about intense, moderate, and low physical activity level during the preceding seven days. Iranians have previously shown the reliability and validity of this questionnaire [19]. The IPAQ calculates everyone’s weekly metabolic equivalent (MET h) of physical activity (Committee, 2005). The surveys were filled out face-to-face during interviews with qualified dietitians. Low physical activity is defined as less than 7.5 MET-hours per day, normal or moderate physical activity is defined as more than 21 MET-hours per day, and high physical activity is defined as more than 21 MET-hours per day.

### Statistical analysis

Data were analyzed using Stata software, version 14.2 (Stata Corp, College Station, TX, USA). essential characteristics of participants across FLI scores were reported as mean ± standard deviation for continuous variables and as percentages for qualitative variables. he Kolmogorov-Smirnov test evaluated the normality of the data. We used the t-test and Chi-square test to compare differences across FLI scores. Binary regression models were applied to determine associations between lifestyle factors and FLI. All statistical analyzes were considered significant according to a P-value of < 0.05 with 95% confidence intervals (CIs).

### Ethical approval

The study was approved by the ethics committee of Kermanshah University of Medical Sciences (KUMS.REC.1394.318). All methods were carried out in accordance with relevant guidelines and regulations. All the participants were provided oral and written informed consent.

## Results

Of the 10,047 participants, only 7,114 were eligible to enroll in the study. From this number, 3687 (51.83%) participants were male, while 3427 (48.17%) were female. the mean ± SD age of the total participants was (45.78 ± 7.80 years). 2017 (28.35%) of the participants had low physical activity (< 7.5 MET-hours per day). Table 1 represents the basic characteristics of the participants based on FLI. The waste circumferences of participants with FLI ≥ 60 were about (105.22 ± 8.03 cm) while participants with FLI < 60 had a lower WC compared to those with FLI ≥ 60 (91.44 ± 8.09 cm) (P < 0.001). Participants with FLI ≥ 60 had higher TG (180.43 ± 91.80 mg/dL) than those with FLI < 60 (105.55 ± 49.05 mg/dL), and this difference was statistically significant (P < 0.001).

**Table 1:** Background characteristics of the study participants according to Fatty Liver Index (FLI) category

Characteristics	All (n = 7,114)	FLI < 60 (n = 4,658)	FLI ≥ 60 (n = 2,456)	*P-*value *
Age (years)	45.78 ± 7.80	45.65 ± 7.97	46.04 ± 7.46	0.04
Sex, n (%)				
Male	3687(51.83)	2416(51.87)	1271(51.75)	0.925
Female	3427(48.17)	2242(48.13)	1185(48.25)	
Residency, n (%)				
Urban	4227(59.42)	2645(56.78)	1582(64.41)	< 0.001
Rural	2887(40.58)	2013(43.22)	874(35.59)	
Marital status, n (%)				
Married	6407(90.06)	4110(88.24)	2297(93.53)	< 0.001
Single	380(5.34)	318(6.83)	62(2.52)	
Divorced and other	92(1.29)	72(1.54)	20(0.81)	
Socio-economic status, n (%)				
Weak	2284(32.11)	1602(34.39)	682(27.78)	< 0.001
Moderate	2342(32.93)	1526(32.76)	816(33.24)	
Good	2487(34.96)	1530(32.85)	957(38.98)	
Physical activity (Met h/day)				
Low	2017(28.35)	1178(25.29)	839(34.16)	< 0.001
Moderate	3340(46.95)	2167(46.52)	1173(47.76)	
High	1757(24.70)	1313(28.19)	444(18.08)	
Alcohol use, n (%)	375 (5.27)	231(4.96)	144(5.86)	0.105
Body Mass Index, kg/m^2^	27.04 ± 4.56	24.91 ± 3.31	31.09 ± 3.82	< 0.001
Waist Hip Ratio	0.93 ± 0.06	0.91 ± 0.05	0.98 ± 0.05	< 0.001
Waist Circumference, cm	96.20 ± 10.39	91.44 ± 8.09	105.22 ± 8.03	< 0.001
Skeletal Muscle Mass, kg	26.83 ± 5.80	25.75 ± 5.40	28.89 ± 5.97	< 0.001
Visceral Fat Area, cm^2^	115.67 ± 50.47	95.31 ± 39.91	154.29 ± 45.42	< 0.001
Fasting blood sugar, mg/dL	89.90 ± 9.49	88.44 ± 8.76	92.67 ± 10.18	< 0.001
Triglycerides, mg/dL	131.40 ± 75.84	105.55 ± 49.05	180.43 ± 91.80	< 0.001
Total cholesterol, mg/dL	183.91 ± 36.86	177.73 ± 35.15	195.62 ± 37.19	< 0.001
Low density lipoprotein-cholesterol, mg/dL	111.20 ± 30.76	108.59 ± 29.79	116.14 ± 31.93	< 0.001
High density lipoprotein-cholesterol, mg/dL	46.46 ± 11.26	48.06 ± 11.38	43.44 ± 10.38	< 0.001
Alanine aminotransferase, IU/L	24.88 ± 15.07	21.94 ± 11.57	30.46 ± 18.89	< 0.001
Aspartate aminotransferase, IU/L	21.50 ± 9.05	20.73 ± 8.14	22.94 ± 10.41	< 0.001
γ-Glutamyl transpeptidase, IU/L	23.61 ± 18.78	18.90 ± 10.18	32.52 ± 26.52	< 0.001
Depression drug use, n%	127(1.79)	75(1.61)	52(2.12)	0.125
Dyslipidemia, n%	2899(40.75)	1446(31.04)	1453(59.16)	< 0.001

Data are shown mean ± SD for continuous variables and n (%) categorical variables. *P- value was obtained t-test and Chi square test

In addition, as the FLI increased, the FBS and T-C increased among the study participants. Between the FLI lower than 60 and higher than 60, there were no statistically significant differences in the prevalence of depression (P = 0.125). Although 127 (1.79%) of the study subjects used depression medications, only 75 (1.61%) were in the FLI 60 groups.

Table 2 provides information about nutritional status based on the FLI among the research participants. Participants with FLI < 60 had a lower daily energy intake (2740.29 ± 970.20 kcal/day) compared to those with FLI ≥ 60 (2840.33 ± 969.47 kcal/day) (P < 0.001). Participants with FLI < 60 had a higher daily carbohydrate intake than those with FLI ≥ 60. Also, daily protein intake in a participant with FLI < 60 was (13.63 ± 2.16%E), while in participants with FLI ≥ 60 was about (13.87 ± 2.10%E), respectively.

**Table 2:** Nutritional status of the study participants according to Fatty Liver Index (FLI) category

Variables	FLI < 60 (n = 4,658)	FLI ≥ 60 (n = 2,456)	*P-*value *
**Energy**			
Energy intake (kcal/d)	2740.29 ± 970.20	2840.33 ± 969.47	< 0.001
Carbohydrate (%E)	61.32 ± 6.22	61.27 ± 5.96	0.735
Protein (%E)	13.63 ± 2.16	13.87 ± 2.10	< 0.001
Fat/oil (%E)	26.94 ± 6.00	26.80 ± 5.66	0.350
**Healthy Eating Index score**			
Quartile 1	1506(32.44)	659(26.88)	< 0.001
Quartile 2	1182(25.46)	643(26.22)	
Quartile 3	1072(23.09)	601(24.51)	
Quartile 4	883 (19.02)	549(22.39)	
**Fat/ Oil**			
Saturated fatty acids, g/da	31.05 ± 15.30	31.76 ± 14.91	0.060
*Trans fatty acids*, g/da	0.322 ± 0.40	0.318 ± 0.37	0.628
n-3 polyunsaturated fatty acids, mg/day	0.047 ± 0.03	0.050 ± 0.03	0.0002
n-6 polyunsaturated fatty acids, mg/day	4.73 ± 3.48	5.12 ± 3.67	< 0.001

*P- value was obtained t-test

The HEI of participants was divided into four quartiles, from highest to lowest score and most of the participants were in the 1st quartile. According to Fat and oil intake among participants, those with FLI ≥ 60 had a higher daily intake of n-3 and n-6 polyunsaturated fatty acids compared to those with FLI < 60 (0.050 ± 0.03 vs. 5.12 ± 3.67; P = 0.001), respectively.

The association between lifestyle factors and fatty liver index according to gender by binary logistic regression analysis is shown in Table 3. In male participants, the risk of NAFLD in good SES was 1.72 (CI 95%: 1.42–2.08) times higher than those with weak SES. Similarly, this value for females was 1.25 (CI 95%: 1.04–1.51). Despite the odds ratio being lower than the unadjusted model after controlling for age, marital status, residency, and alcohol use, the association between SES and NAFLD remained significant.

**Table 3:** Association of lifestyle factors on fatty liver index in binary logistic regression analysis

FLI ≥ 60	Male				Female			
	Model 1		Model 2		Model 1		Model 2	
	Odds ratio	P value	Odds ratio (95% CI)	P value	Odds ratio (95% CI)	P value	Odds ratio (95% CI)	P value
Socio-economic status								
Weak	Ref.		Ref.		Ref.		Ref.	
Moderate	1.21(0.99–1.49)	0.057	1.18(0.96–1.45)	0.110	1.38(1.17–1.62)	< 0.001	1.28(1.08–1.53)	0.004
Good	1.72(1.42–2.08)	< 0.001	1.63(1.33-2.00)	< 0.001	1.25(1.04–1.51)	0.015	1.17(0.95–1.45)	0.133
Physical activity (Met h/day)								
Low	Ref.		Ref.		Ref.		Ref.	
Moderate	0.74(0.63–0.88)	0.001	0.76(0.64–0.89)	0.001	0.81(0.69–0.96)	0.016	0.77(0.65–0.91)	0.003
High	0.43(0.37–0.51)	< 0.001	0.44(0.37–0.53)	< 0.001	0.53(0.41–0.70)	< 0.001	0.54(0.41–0.71)	< 0.001
Visceral Fat Area	1.05(1.05–1.18)	< 0.001	1.05(1.01–1.45)	< 0.001	1.05 (1.04–1.05)	< 0.001	1.05(1.04–1.05)	< 0.001
Energy intake (kcal/d)	1.00(1.00-1.23)	0.001	1.00(1.00-1.19)	0.019	1.00 (1.00-1.51)	0.045	1.00(1.00-1.09)	0.009
Depression	0.89(0.49–1.62)	0.711	0.93(0.51–1.70)	0.822	1.68(1.06–2.64)	0.025	1.71(1.07–2.71)	0.022
Dyslipidemia	4.13(3.57–4.80)	< 0.001	4.09(3.52–4.75)	< 0.001	2.81(2.41–3.27)	0.002	2.69(2.31–3.14)	0.003

Model 1: Unadjusted model

Model 2: adjusted for age, marital status, residency and alcohol use

*Obtained by binary logistic regression, P value < 0.05

The odds of NAFLD in males with high physical activity was significantly 56% (OR: 0.44, 95% CI: 0.37–0.53), and in females, 46% (OR: 0.54, 95% CI: 0.41–0.71) less than males and female with low physical activity (P < 0.001). Increasing daily energy intake was associated with a significant increase in the odds of NAFLD in males (P = 0.019) and females (P = 0.009).

The odds of NAFLD in female participants with depression were 71% higher than in non-depressed participants (OR:1.71; CI 95%: 1.07–2.71). This association was not significant in males.

## Discussion

The current study aimed to investigate the association between lifestyle risk factors and fatty liver index in Iranian adults. According to our knowledge, this is the first study that examines the impact of biomarkers and lifestyle in this large population, known as Kurdish, who have a different culture and lifestyle from another part of the country. our study found, that good SES, high VFA, and dyslipidemia were associated with an increased risk of NAFLD. Conversely, high physical activity reduces the risk of NAFLD.

Regardless of alcohol use, NAFLD is a prevalent metabolic condition known for the buildup of extra fat in the liver cells. NAFLD is associated with dyslipidemia, obesity, and insulin resistance [20]. The results of our research showed that high fatty liver scores had a positive association with WC. A similar finding, by Rocha et al., revealed that BMI and WC had the highest prevalence rates among NAFLD patients. Thus, they suggested WC as the greatest predictor for NAFLD [21]. Also, in a recent meta-analysis, the incidence of NAFLD was significantly elevated by rising BMI and WC [22]. Our findings showed statistically significant differences between the incidence of NAFLD and blood biomarkers such as TG, TC, LDL-C, HDL-C, and FBG when comparing subjects with an FLI < 60 vs. FLI ≥ 60. For instance, participants with high fatty liver scores had higher FBG levels than those with low fatty liver scores. According to recent research by Marchesini et al., NAFLD patients had greater levels of insulin resistance than controls, as measured by HOMA-IR [23]. This conclusion is consistent with Sathiaraj et al. reporting, who discovered that patients with NAFLD had significantly higher mean TC, low HDL-C, and TG levels than healthy controls [24]. In addition, the presence of insulin resistance is defined as baseline FBG values between 110 and 126 mg/dl and two of the following factors: arterial hypertension, triglycerides above 150 mg/dl and/or HDL-C < 35 in males and < 39 in females, a waist/hip index > 0.90 in males and 0.85 in females and/or BMI > 30 kg/m2, and alanine aminotransferase > 40 proposed that this subject has a high probability of developing NAFLD[25].

The key risk factors for NAFLD, such as insulin resistance, obesity, and lipid metabolic disease, have been specifically linked to SES [26]. In this area data is controversial. Goodman et al. reported that those with lower SES had a greater BMI and more insulin resistance than people with higher SES [27]. Furthermore, Cho et al. found a connection between SES inequalities and an increase in the risk of NAFLD, demonstrating how SES might negatively impact the main risk factors for NAFLD. Our data revealed that participants with a good SES had substantially higher FLI scores than those in the medium and weak SES groups. In line with our study, Tomah et al. in 2021 [28] observed that higher SES was associated with increased severity of steatosis. These findings suggest a relationship between SES inequalities and the prevalence of NAFLD, probably due to more significant risk variables.

Another conclusion in the current research is that the PA level was considerably lower in the subjects with high fatty liver scores than in the subjects with lower fatty liver scores. Previous studies revealed that NAFLD is positively linked to sedentary and lower PA levels [29]. Other studies showed that patients with NAFLD could improve their hepatic lipid metabolism by reducing the accumulation of hepatic fat, increasing insulin sensitivity, and increasing muscle mass without needing to lose weight by engaging in low- and moderate-intensity aerobic exercise and consistent resistance training three times per week for eight weeks [30]. The relationship between active PA and a lower risk of developing NAFLD was emphasized by a comprehensive review and meta-analysis that evaluated these effects [31]. They concluded that exercising both aerobically and anaerobically for four months reduced liver fat content, BMI, adipose tissue, and insulin resistance [31]. When NAFLD ORs were calculated using PA, those with moderate physical activity had considerably more significant ORs than those with high physical activity.

The nutritional status of a participant in our study showed that people who had FLI < 60 follow a healthier eating pattern (higher healthy eating index) than those who had FLI > 60 and this difference was statically significant. Numerous observational studies have investigated the relationship between food habits and NAFLD. In a cross-sectional study with 170 NAFLD patients in Iran, a healthy dietary pattern identified by factor analysis was connected with a lower risk of fibrosis, whereas a Western pattern was associated with a higher risk of fibrosis [32]. A factor analysis-derived fast food-type pattern was linked to a greater risk of NAFLD in a Greek case-control study [33]. A cross-sectional investigation in a Chinese population using specified dietary indicators revealed that the Diet Quality Index-International was linked to a decreased prevalence of NAFLD [34]. Preferable eating patterns and high-quality meals were supported mainly by this observational research, either cross-sectional or case-control studies. NAFLD has a complex etiology that certainly includes interactions between genetic and environmental variables [35]. Certain nutritional components, such as fiber, monounsaturated and omega-3 fatty acids, and phytosterols, have anti-inflammatory and antioxidant properties and are among the many hypothesized pathways relating diet quality to NAFLD risk [36]. Diet may also affect the onset of NAFLD by altering body weight and metabolic syndrome [36]. There have also been reports of possible correlations between genetic NAFLD susceptibility and the food quality [37].

In females, depression defined with medication use was associated with higher odds of NAFLD. According to Elwing et al., those who had nonalcoholic steatohepatitis had considerably higher PHQ-9 scores [38]. In contrast, a case-control study by Blaga et al. found no connection between depression and NAFLD [39]. These contradictory findings suggested that an unknown independent component may cause depression in NFLD patients. Reactive oxygen and nitrogen species, which cause increased oxidative stress on the antioxidant defense system, are linked to the genesis of depression [40]. The failure of the protective antioxidant system, which may result in NAFLD, may be caused by an imbalance between decreasing antioxidant levels and higher oxidative stress. Also, a point seen in Table 2, is the higher consumption of omega-3 in the group with a high fatty liver index. According to previous studies [41], the consumption of omega-3 is effective in improving the fatty liver, still in the case of the present study, it can be seen that in both groups, the amount of intake is lower than the therapeutic dose.

Our study’s high sample size is its key strength. This study had several drawbacks, including the fact that it was cross-sectional, which prevented it from concluding causation. Additional research is required. Second, there is some recall bias present in FFQ. However, we employed the validated dietary questionnaire to calculate the HEI; as a result, face-to-face interviews were used to evaluate diet, potentially reducing measurement error. Although we are unable to evaluate changes in the diet over time, longitudinal studies analyzing these correlations are required to establish causation. Finally, the lack of data related to MRI, a significant limitation is the lack of liver biopsy data, which is the gold standard for NAFLD diagnosis.

## Conclusion

In our study, we found that good SES, high VFA, and dyslipidemia were associated with an increased risk of NAFLD. Conversely, high physical activity reduces the risk of NAFLD. Therefore, lifestyle modification may help to improve liver function.

## Data Availability

The data analyzed in the study are available from the corresponding author upon reasonable request.
